# Supplementary figures and images for: Cyclooxygenase production of PGE2 promotes phagocyte control of A. fumigatus hyphal growth in larval zebrafish
Source: PLoS Pathog. 2022 Mar 25;18(3):e1010040. doi: 10.1371/journal.ppat.1010040 (PMC8986117; doi:10.1371/journal.ppat.1010040)

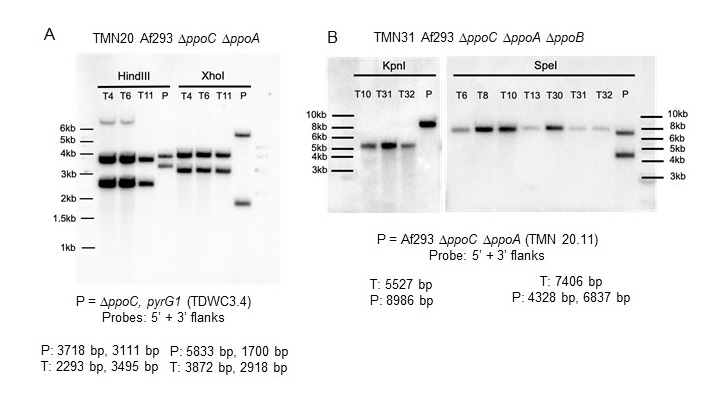

Supplement: S2 Fig — Confirmation of (A) TMN20 Af293 ΔppoC ΔppoA double mutant and (B) TMN31 Af293 ΔppoC ΔppoA ΔppoB triple mutant. Restriction enzyme digestion, southern blotting and hybridization were performed as mentioned in the Materials and Methods. Double and triple ppo mutants were created in sequence. Hybridization of αP32-dCTP labeled 5´ and 3´ flank regions were used to confirm transformants. The parental strain and the size of DNA fragments used to probe for southern blotting and hybridization are shown in each figure. P = parental strain; T = transformants. (TIF) [file ppat.1010040.s002.tif]

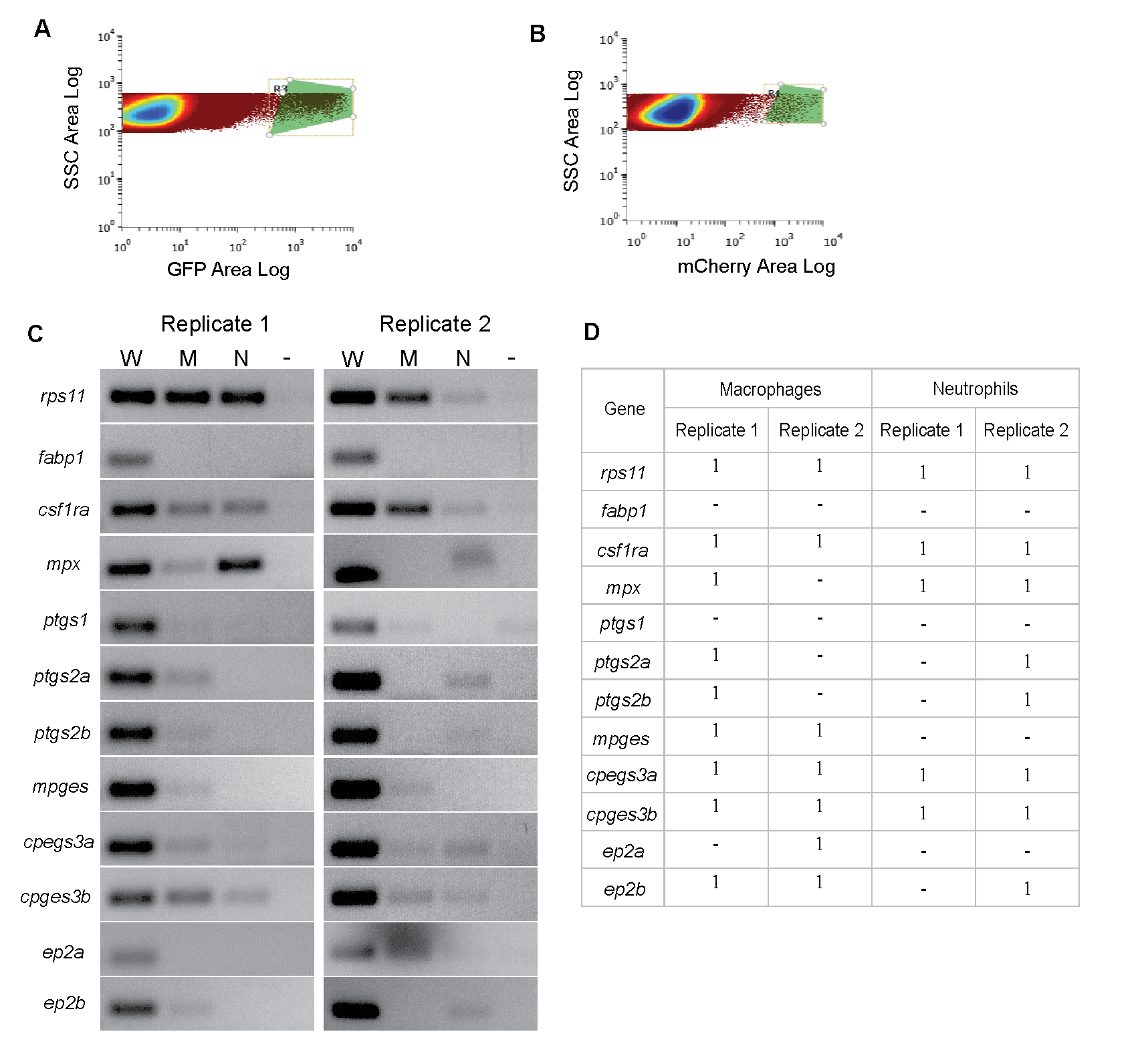

Supplement: S4 Fig — Larvae expressing GFP in macrophages (Tg(mpeg1:H2B-GFP)) or mCherry in neutrophils (Tg(mpx:mCherry)) were trypsinized and single cell suspensions were subjected to FACS. Two independent replicates were performed. (A, B) Gating used to isolate GFP-expressing macrophages (A) and mCherry expressing neutrophils (B). (C, D) RNA was extracted from sorted cells and RT-PCR was performed for the genes shown. (C) DNA gels for two replicates. W: whole larvae control, M: isolated macrophages, N: isolated neutrophils, -: negative controls. (D) Summary table of gene expression from both replicates. Presence of a band is represented by 1. (TIF) [file ppat.1010040.s004.tif]

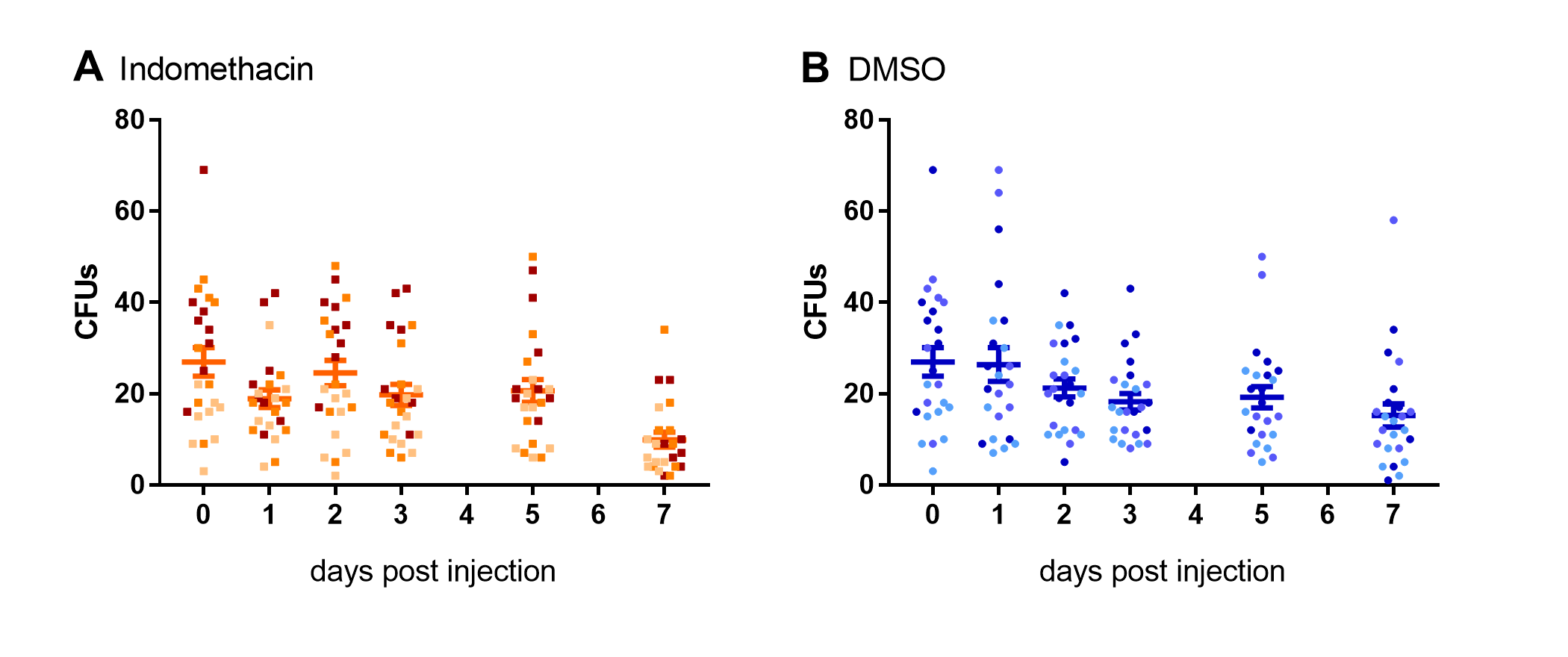

Supplement: S5 Fig — Wild-type larvae were injected with TBK1.1 (Af293) spores at 2 dpf, exposed to (A) 10 μM indomethacin or (B) DMSO vehicle control, and fungal burden was quantified by homogenizing and plating individual larvae for CFUs at multiple days post injection. Eight larvae per condition, per dpi, per replicate were quantified, and the number of CFUs at each dpi is represented. Each data point represents an individual larvae, color-coded by replicate. Bars represent means ± SEM from three individual replicates. Average injection CFUs: 27. This same data is normalized to the initial spore injection and presented in Fig 4. (TIF) [file ppat.1010040.s005.tif]

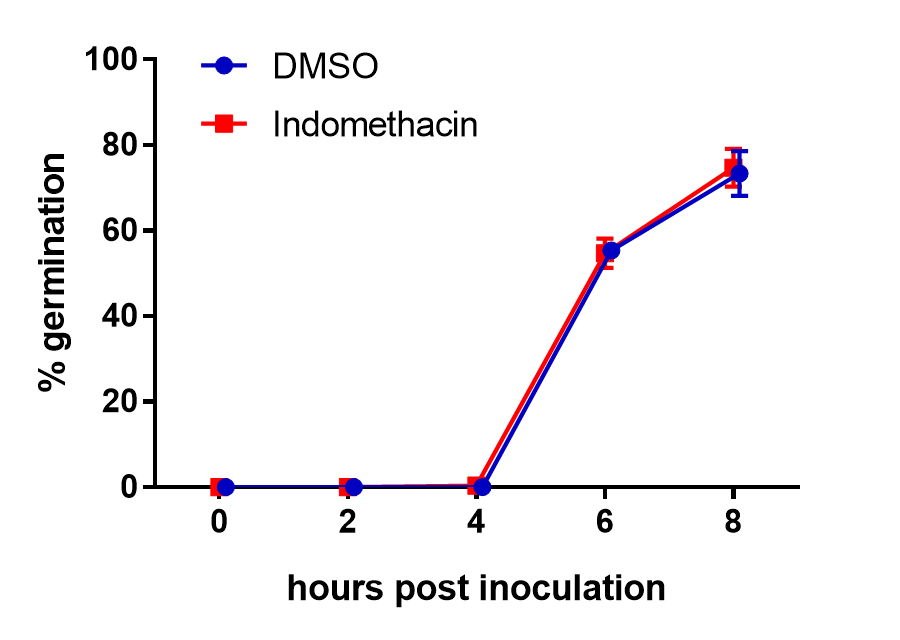

Supplement: S6 Fig — TBK1.1 (Af293) spores were inoculated into RPMI media in the presence of 10 μM indomethacin or DMSO vehicle control. Every 2 hours, an aliquot of spores was removed and scored for germination. Points represent pooled means ± SEM from two independent replicates. (TIF) [file ppat.1010040.s006.tif]

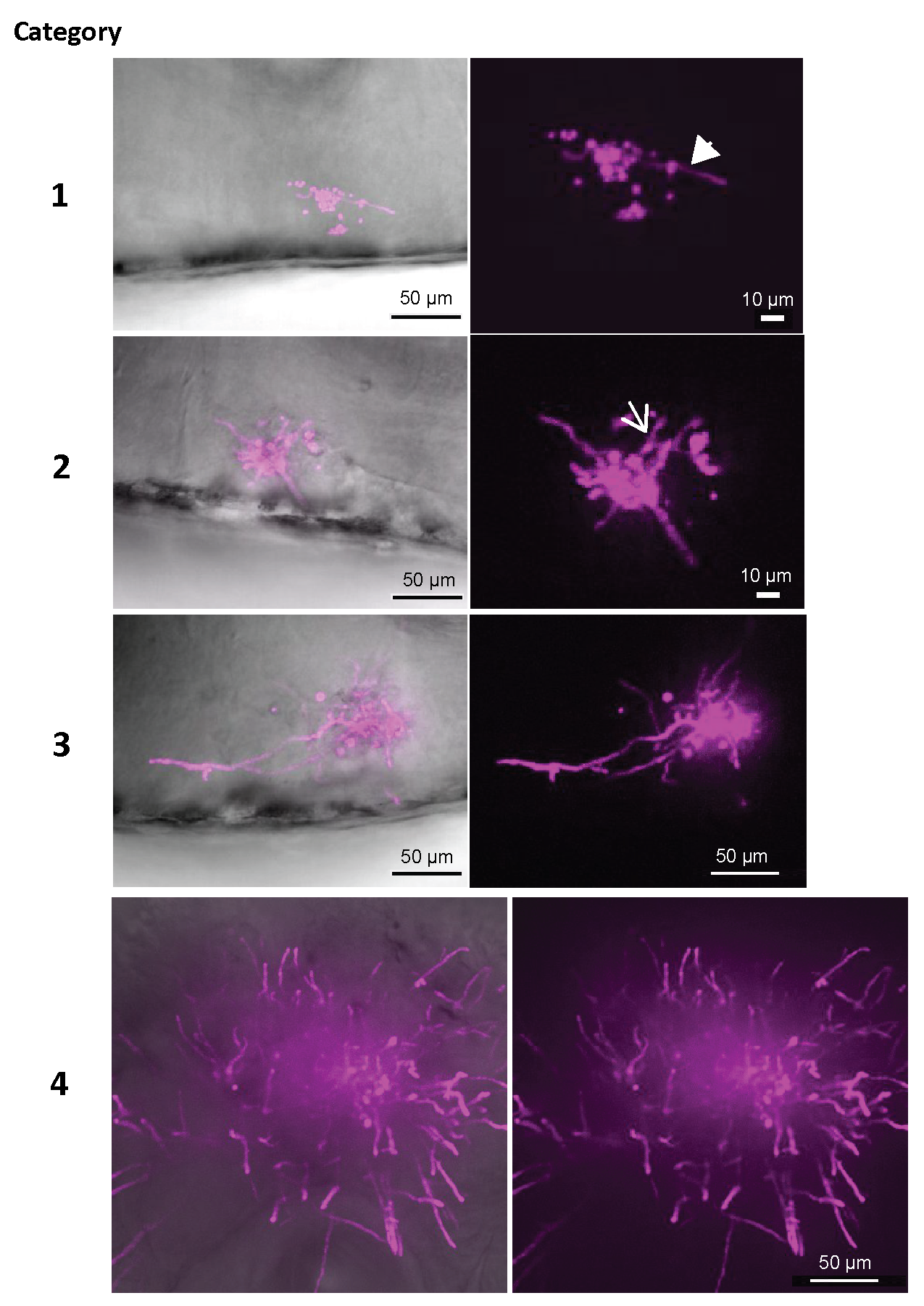

Supplement: S7 Fig — Wild-type larvae were injected with mCherry-expressing TBK5.1 (Af293) spores, exposed to 10 uM indomethacin or DMSO vehicle control and imaged at 1, 2, 3, and 5 dpi. Incidences of hyphal growth were scored a value of 1–4 depending on the extent of hyphae. Category 1: presence of one germ tube (white arrow). Category 2: presence of branched hyphae (open white arrow), yet small fungal bolus. Category 3: presence of spread-out invasive hyphae. Category 4: presence of severe invasive hyphae and tissue damage. Scale bars = 50 μm or 10 μm. (TIF) [file ppat.1010040.s007.tif]

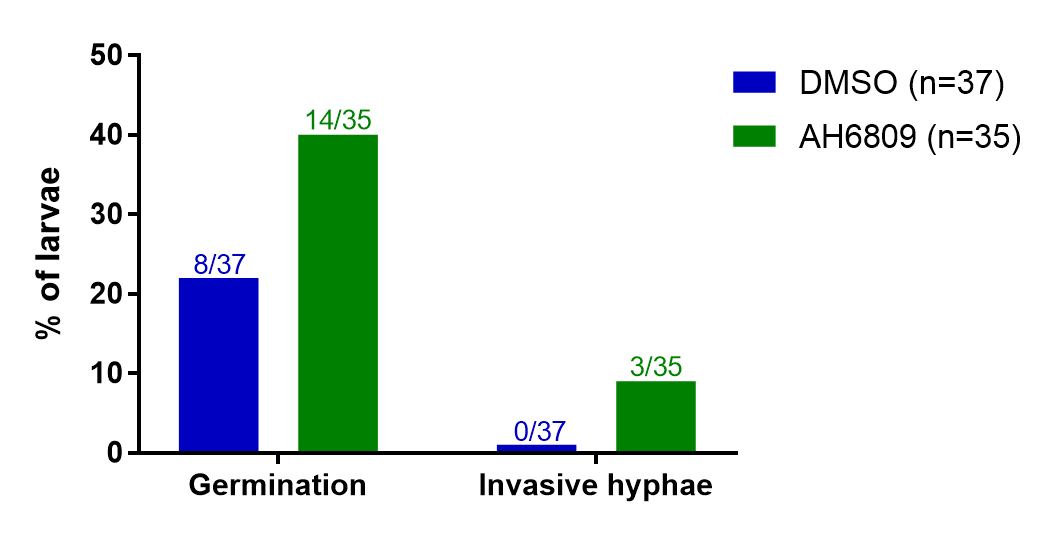

Supplement: S8 Fig — Wild-type larvae were injected with mCherry-expressing TBK5.1 (Af293) spores and exposed to 5 μM AH6809 or DMSO vehicle control at 2 dpf. Larvae were imaged at 3 dpi and the percentage of larvae with germination and invasive hyphae was calculated. Data are pooled from three independent replicates, at least 11 larvae per condition, per replicate, P values calculated by Fisher’s Exact Test. (TIF) [file ppat.1010040.s008.tif]

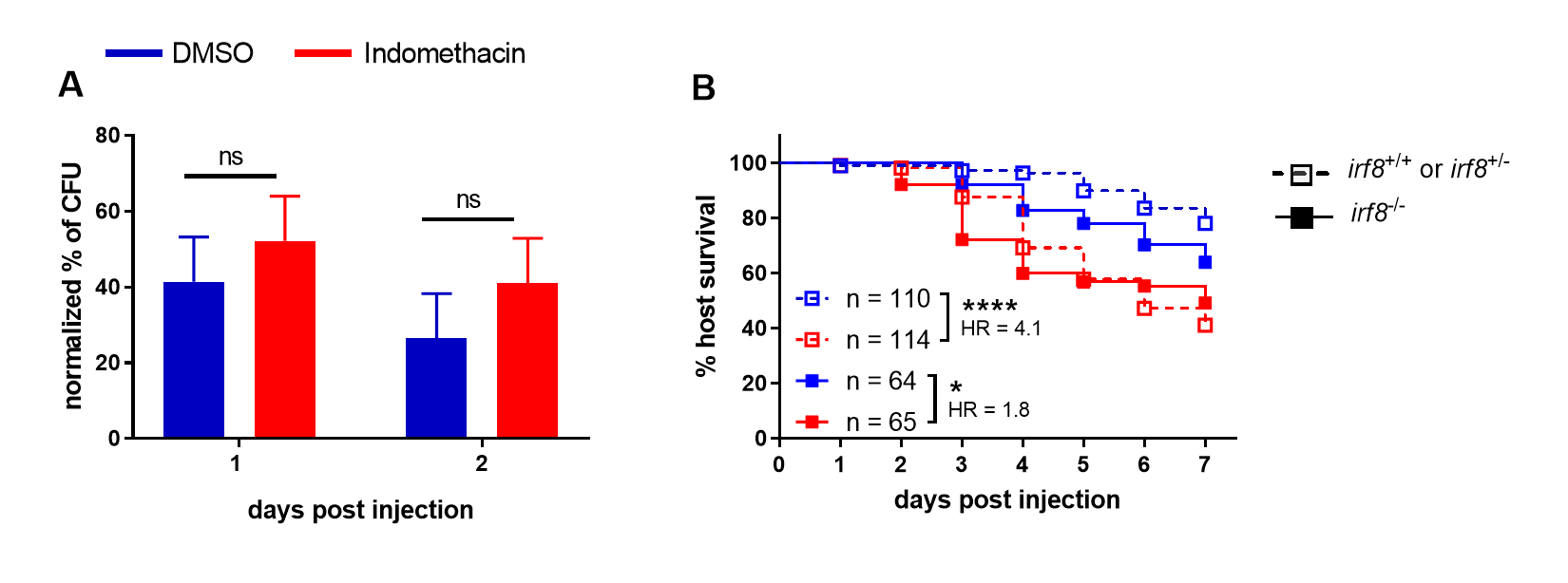

Supplement: S9 Fig — Macrophage-deficient irf8-/- or control (irf8+/+ or irf8+/-) larvae were injected with A. fumigatus TFYL49.1 (CEA10) strain and treated with 10 μM indomethacin or DMSO vehicle control. (A) Fungal burden was monitored by homogenizing individual larvae and quantifying CFUs at 1 and 2 dpi. CFUs from irf8-/- were normalized to CFUs of irf8+/+/irf8+/- at each dpi for each condition. Data were pooled from four independent replicates, 8 larvae per condition, per dpi, and P values calculated by ANOVA. (B) Larvae were monitored for survival. Data are pooled from five independent replicates, at least 8 larvae per condition, per replicate and the total larval N per condition is indicated. Cox proportional hazard regression analysis was used to calculate P values and hazard ratios (HR). Average injection CFUs: irf8+/+/irf8+/- = 26, irf8-/- = 20. (TIF) [file ppat.1010040.s009.tif]

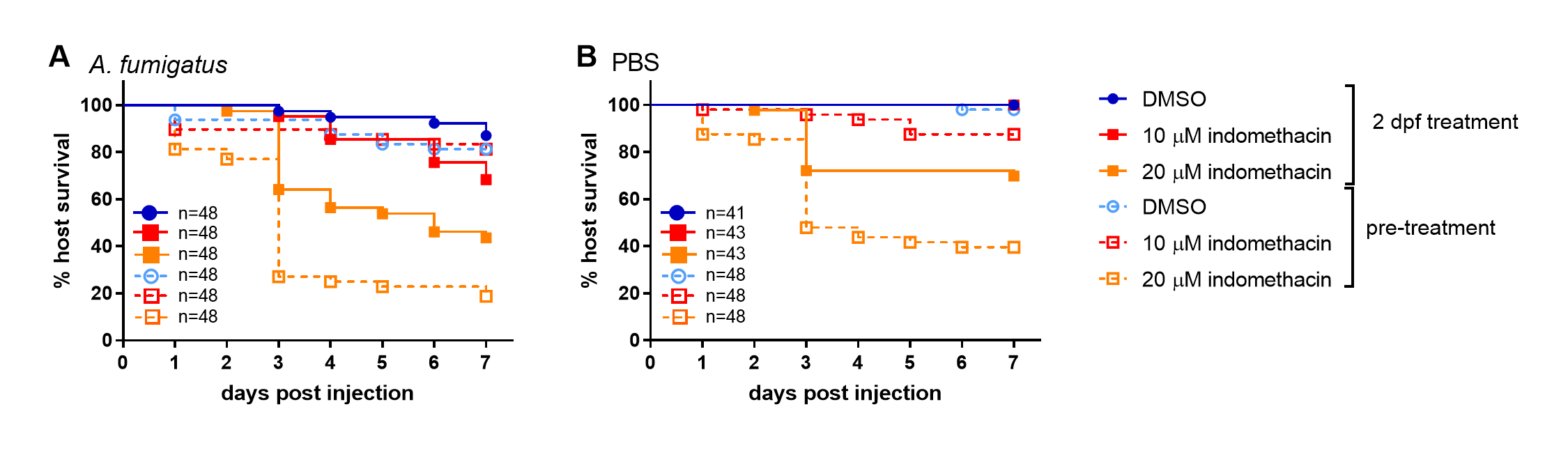

Supplement: S10 Fig — Larvae were exposed to 10 μM or 20 μM indomethacin, or DMSO vehicle control at 1 dpf (1 day prior to injection) or at 2 dpf (immediately after injection) and survival was monitored for 7 days. Survival of larvae injected with (A) TBK1.1 (Af293) A. fumigatus spores, or (B) PBS mock-infection. Data are pooled from two independent experiments, at least 20 larvae per condition, per replicate and the total larval N per condition is indicated in each figure. (A) Average injection CFUs: 2 dpf treatment = 35, pre-treatment DMSO = 38, 10 μM indomethacin = 50, 20 μM indomethacin = 62. (TIF) [file ppat.1010040.s010.tif]

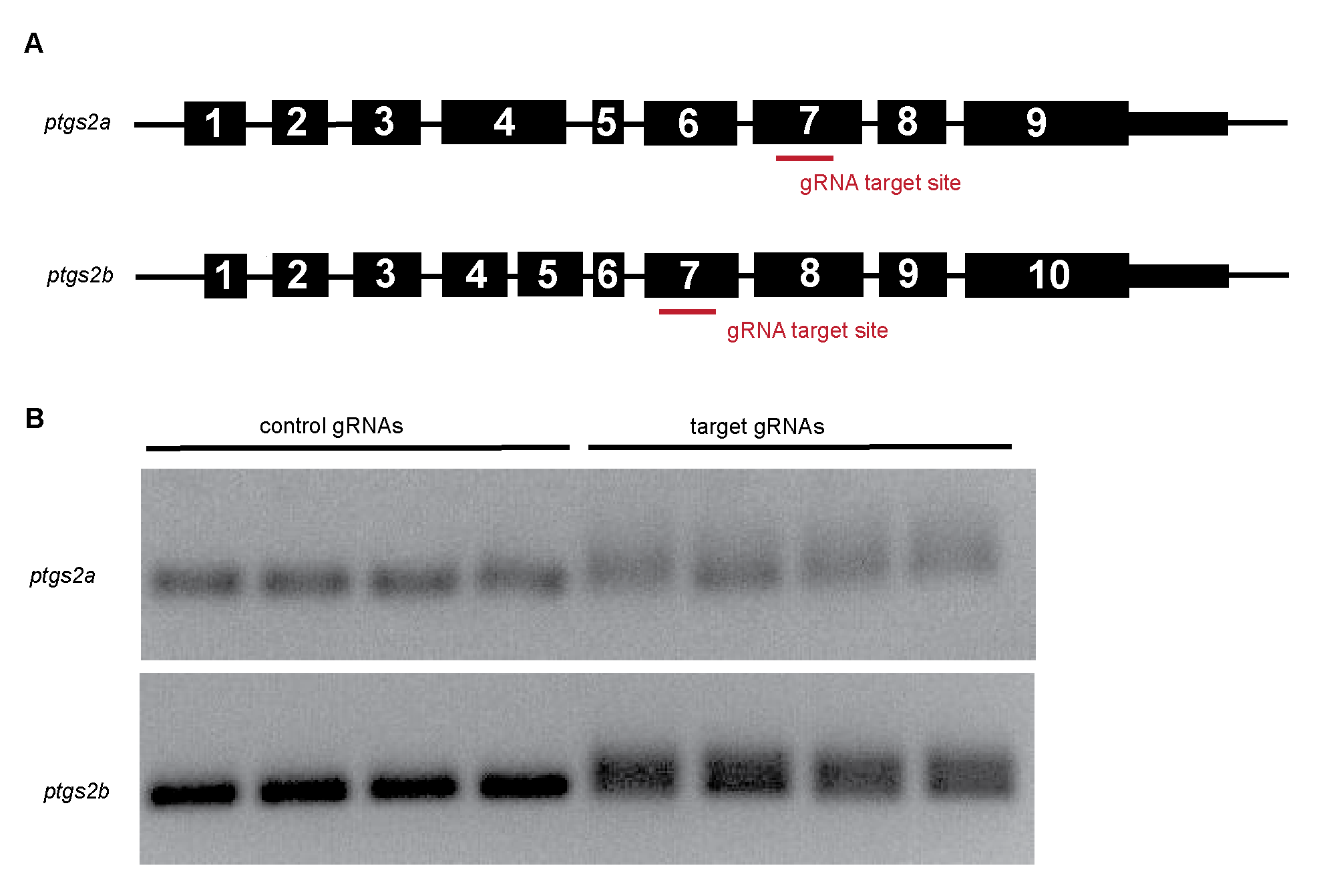

Supplement: S11 Fig — (A) A schematic showing the structure of ptgs2 genes and the target sites for gRNA binding. (B) Wild-type larvae were injected with gRNAs targeting both ptgs2a and ptgs2b or gfp control. Genomic DNA from 4 individual larvae from each group was used in PCR reactions with primers flanking the gRNA target sites. Each lane represents PCR amplification of an individual larva. (TIF) [file ppat.1010040.s011.tif]

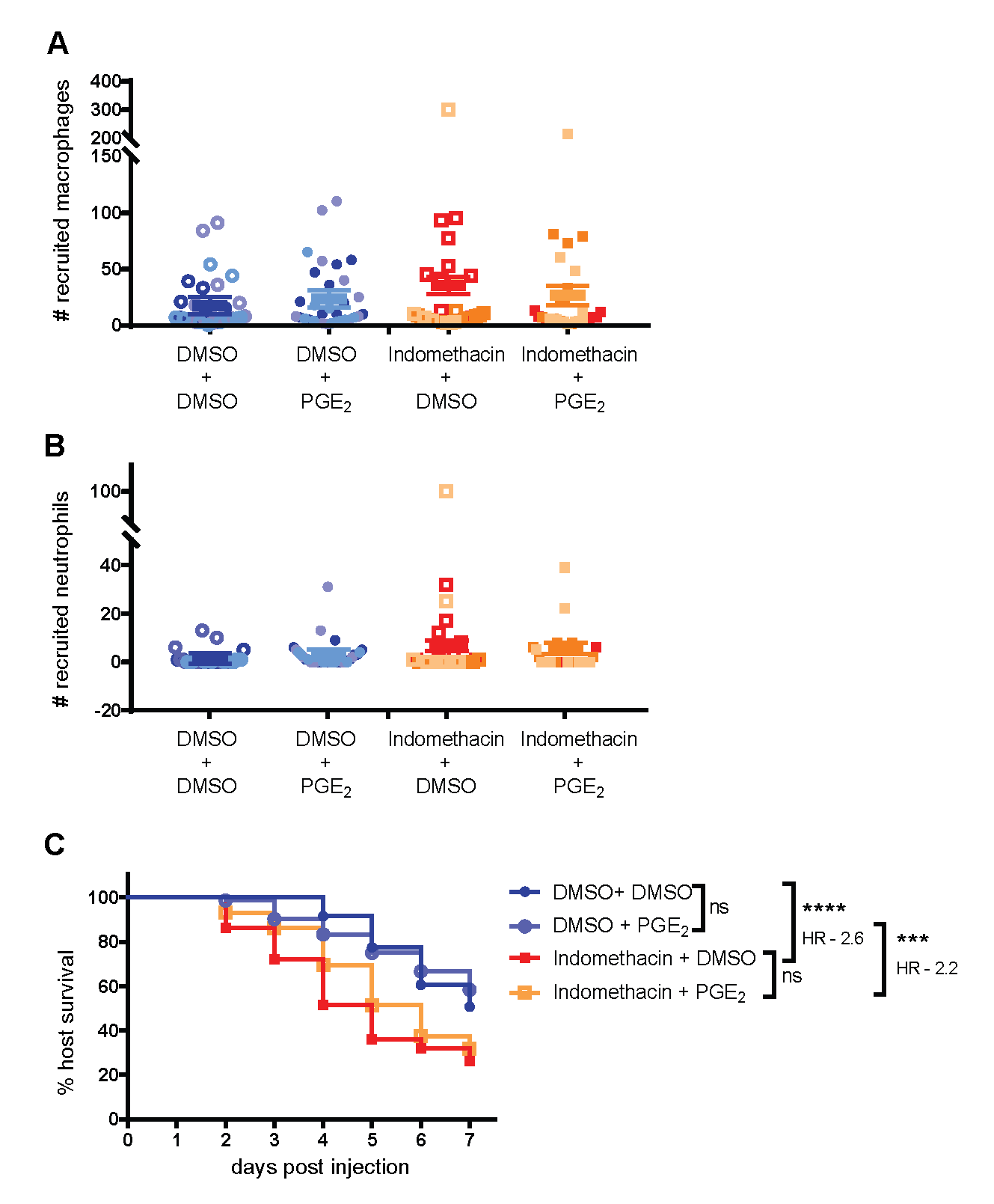

Supplement: S12 Fig — At 2 dpf, larvae were injected with TBK5.1 (Af293), followed by injection of 10 μM PGE2 or DMSO vehicle control at 3 dpf (1 dpi). (A, B) Macrophage nuclear-labeled Tg(mpeg1:H2B-GFP) and neutrophil-labeled Tg(lyz:BFP) larvae were imaged at 3 dpi and the number of (A) macrophages and (B) neutrophils were enumerated. Data are pooled from three independent replicates, at least 8 larvae per condition, per replicate. Each data point represents an individual larva, color-coded by replicate. Bars represent pooled emmeans ± SEM and P values were calculated by ANOVA. (C) Survival of injected and treated neutrophil-defective larvae (mpx:rac2D57N) was monitored. Cox proportional hazard regression analysis was used to calculate P values and hazard ratios (HR). Data are pooled from three replicates, at least 23 larvae per condition, per replicate. Average injection CFUs: 49. (TIF) [file ppat.1010040.s012.tif]
