# Supplementary material for: Cyclooxygenase production of PGE2 promotes phagocyte control of A. fumigatus hyphal growth in larval zebrafish
Source: PLoS Pathog. 2022 Mar 25;18(3):e1010040. doi: 10.1371/journal.ppat.1010040 (PMC8986117; doi:10.1371/journal.ppat.1010040)
Supplement: S1 Table — (DOCX) [file ppat.1010040.s013.docx]

**S1 Table. Primers used in this study.**

|  | Name | Sequence (5´-3´) | Purpose | Reference |
| --- | --- | --- | --- | --- |
| Primers used to construct *A. fumigatus ∆ppo* triple mutant strain | GF ppoA del Cassette F | CGCGCGTAATACGACTCACTATAGG | *ppoA* deletion cassette | This study |
|  | GF ppoA del Cassette R | AACAAAAGCTGGAGCTCCACC | *ppoA* deletion cassette |  |
|  | MN KOppoA 5´ flank F | GGCGTCGATTTGCTTATCTAAGC | *ppoA* deletion 5´ flank forward for southern analysis |  |
|  | MN KOppoA 5´ flank R | CGACAGCAACTCATGAGGAGG | *ppoA* deletion 5´ flank reverse for southern analysis |  |
|  | MN KOppoA 3´ flank F | GTGACTCGACTGTATGGAGTAG | *ppoA* deletion 3´ flank forward for southern analysis |  |
|  | MN KOppoA 3´ flank R | GATCCAATACATCCACCATGTGC | *ppoA* deletion 3´ flank reverse for southern analysis |  |
|  | MN ppoA ORF F | TCTTCACGGAGTCAGAGTTGTACC | PCR screen of *ppoA* forward |  |
|  | MN ppoA ORF R | CTTTACTTCTGGCAAATCGCCATCC | PCR screen of *ppoA* reverse |  |
|  | MN KOppoB 5´ flank F | TGGTGCCTCGTGACAAGTC | *ppoB* deletion 5´ flank forward |  |
|  | MN KOppoB 5´ flank R | CTCTATTGACCTATAGGACCTGAGTGATGCCCAAATGACTGGAGCAACCC | *ppoB* deletion 5´ flank reverse |  |
|  | MN KOppoB 3´ flank F | TTAAGTTGAGCATAATATGGTCCATCTAGTGCGGGTGCATTACGTACAAG | *ppoB* deletion 3´ flank forward |  |
|  | MN KOppoB 3´ flank R | GCTGAACTGGACTGCCTC | *ppoB* deletion 3´ flank reverse |  |
|  | MN ppoB ORF F | GCAATGGCAGTGATCTTCACAGC | PCR screen of *ppoB* forward |  |
|  | MN ppoB ORF R | AATGTCCCGGCACATTACATACG | PCR screen of *ppoB* reverse |  |
| Primers used to PCR CRISPR target sites | ptgs2a F | TCTTCAGTGAGCGTTTCCAG | PCR *ptgs2a* CRISPR target site | This study |
|  | ptgs2a R | TCCTGGATCTGAAAGTTGTCC |  |  |
|  | ptgs2b F | TGAATCTCGTCGATTTGCTG | PCR *ptgs2b* CRISPR target site |  |
|  | ptgs2b R | CAGCCTCTCATCATCCCAGT |  |  |
| Primers used to test gene expression using RT-PCR | qrps11 F | TAAGAAATGCCCCTTCACTG | RT-PCR *rps11* | [1] |
|  | qrps11 R | GTCTCTTCTCAAAACGGTTG |  |  |
|  | qfabp1 F | AGGGGATTGAGTCTGTGACG | RT-PCR *fabp1* | This study |
|  | qfabp1 R | CATGAGATGCGTCTGCTGAT |  |  |
|  | qcsf1ra F | ATGTCCAGACCTGACTTCGC | RT-PCR *csf1ra* | [2] |
|  | qcsf1ra R | TCGGATGTTTCTCCCAGCAT |  |  |
|  | qmpx F | AGGTGTTGCTGAGCCTTTTG | RT-PCR *mpx* | [2] |
|  | qmpx R | TGGTGAACACTCCATTGCTC |  |  |
|  | qptgs1 F | CAGCCGTTCAATGAATATCG | RT-PCR *ptgs1* | This study |
|  | qptgs1 R | CCGTATAGTTCCTCCAGCTCTT |  |  |
|  | qptgs2a F | TGCATACAGAAGACGCTTCAA | RT-PCR *ptgs2a* | This study |
|  | qptgs2a R | CGTGACCGTACAGCTCCTTC |  |  |
|  | qptgs2b F | CAGGAAACGCTTCAACATGA | RT-PCR *ptgs2b* | This study |
|  | qptgs2b R | CTCCACAGCATCCACATCTC |  |  |
|  | qmpges F | CGTTTGCTAACCCAGAGGAC | RT-PCR *mpges* | This study |
|  | qmpges R | GGGCAAAATGTTCTCCATGT |  |  |
|  | qcpges3a F | ACAACATGGGAGGGGAAGAT | RT-PCR *cpges3a* | This study |
|  | qcpges3a R | GGCATGACAGATCCTGTTCC |  |  |
|  | qcpges3b F | TCCAACTATGATCGCTTTTCA | RT-PCR *cpges3b* | This study |
|  | qcpges3b R | GCTTGCCAGTTGCTTATTCC |  |  |
|  | qep2a F | ATGCAAAATTTCCCTTAGGC | RT-PCR *ep2a* | This study |
|  | qep2a R | GCCCACAATAGCTGCTCAAT |  |  |
|  | qep2b F | AAGAGACACCGCTCCATGAC | RT-PCR *ep2b* | This study |
|  | qep2b R | TGTGACGATCTCGGTGGTAG |  |  |

**References**

1. Oliveira E, Casado M, Raldua D, Soares A, Barata C, Pina B. Retinoic acid receptors' expression and function during zebrafish early development. The Journal of steroid biochemistry and molecular biology. 2013;138:143-51.

2. Rosowski EE, Deng Q, Keller NP, Huttenlocher A. Rac2 Functions in Both Neutrophils and Macrophages To Mediate Motility and Host Defense in Larval Zebrafish. Journal of immunology. 2016;197(12):4780-90.
